# DNA methylation-mediated silencing of PU.1 in leukemia cells resistant to cell differentiation

**DOI:** 10.1186/2193-1801-2-392

**Published:** 2013-08-21

**Authors:** María José Fernández-Nestosa, Estefanía Monturus, Zunilda Sánchez, Francisco S Torres, Agustín F Fernández, Mario F Fraga, Pablo Hernández, Jorge B Schvartzman, Dora B Krimer

**Affiliations:** Department of Cellular and Molecular Biology, Centro de Investigaciones Biológicas (CSIC), Ramiro de Maeztu 9, 28040 Madrid, Spain; Scientific and Applied Computing Laboratory, Facultad Politécnica, Universidad Nacional de Asunción, San Lorenzo, Paraguay; Laboratory of Molecular Biology, Instituto de Investigaciones en Ciencias de la Salud, Universidad Nacional de Asunción, San Lorenzo, Paraguay; Cancer Epigenetics Laboratory, Instituto Universitario de Oncología del Principado de Asturias (IUOPA), Universidad de Oviedo, Asturias, Spain; Centro Nacional de Biotecnología/CNB-CSIC, Madrid, Spain

**Keywords:** PU.1, Erythroleukemia cells, SFFV, DNA methylation

## Abstract

In mice, the proviral integration of the Friend Spleen Focus Forming Virus (SFFV) within the PU.1 locus of erythroid precursors results in the development of erythroleukemia. SFFV integrates several kilobases upstream of the PU.1 transcription initiation start site leading to the constitutive activation of the gene which in turn results in a block of erythroid differentiation. In this study we have mapped and sequenced the exact location of the retroviral integration site. We have shown that SFFV integrates downstream of a previously described upstream regulatory element (URE), precisely 2,976 bp downstream of the URE-distal element. We have also found that SFFV persists integrated within the same location in resistant cell lines that have lost their differentiation capacity and in which case PU.1 remains silent. We have examined the methylation status of PU.1 and found that in resistant cells the nearby CpG islands remained methylated in contrast to a non-methylated status of the parental cell lines. Treatment with 5-aza-2′-deoxycytidine caused resistant cells to differentiate yet only when combined with HMBA. Altogether these results strongly suggest that methylation plays a crucial role with regard to PU.1 silencing. However, although demethylation is required, it is not sufficient to overcome the differentiation impasse. We have also showed that activation blockage of the Epo/Epo-R pathway remains despite of the absence of PU.1.

**Electronic supplementary material:**

The online version of this article (doi:10.1186/2193-1801-2-392) contains supplementary material, which is available to authorized users.

## Introduction

PU.1, the ETS transcription factor encoded by the Sfpi/PU.1 gene, is crucial for the regulation of hematopoietic development (Burda et al. 2010). The gene is already expressed at high levels in multipotential progenitors, including erythroblasts. Thereafter, PU.1 expression is tightly regulated and in mature hematopoietic cells is maintained in myeloid and B lymphoid cells but not in erythrocytes or T cells. Like other transcription factors and proteins, PU.1 can posses dual roles and play as an activator or repressor depending on its combination with variable binding-partners of each specific cell lineage. PU.1 holds a tumor suppresor activity during myeloid leukemia by promoting maturation of myeloid cells but acts as an oncogene when overexpressed in proerythroblasts by disrupting the erythroid differentiation program (Cook et al. 2004; Moreau-Gachelin et al. 1996; Rosenbauer et al. 2004).

Immortalized murine erythroleukemia (MEL) cells which are derived from transformed erythroblasts by the Friend complex virus constitute an appropriate and valuable model to study tumor cell reprogramming (Moreau-Gachelin 2006; Papetti and Skoultchi 2007). Insertion of the Friend spleen focus-forming virus (SFFV) upstream of the transcription start site of PU.1 leads to constitutive gene expression and to blockage of erythroid cell differentiation (Ruscetti 1999). MEL cells can be induced to reinitiate the differentiation program by the addition of chemical agents such as HMBA, in which case down regulation of PU.1 has been shown to be a critical event that takes place during early cell differentiation (Rao et al. 1997). We have previously reported on the establishment of HMBA-resistant cell lines (MEL-R) in which PU.1 remains silent even though the SFFV persists integrated within a similar location to the site found in MEL parental lines (Fernández-Nestosa et al. 2008). In this study, we have further characterized the concrete location of the SFFV integration site in both, parental and resistant cells, to confirm that no further changes have occurred in the resistant clones. The present analysis has revealed that the SFFV integration position in parental and resistant MEL cell lines is located downstream of the upstream regulatory element (URE) (Okuno et al. 2005), concretely at 2,976 bp from the distal element.

We have also studied the methylation status of the PU.1 promoter and determined that the four CpG islands close to the PU.1 promoter remain methylated in MEL-resistant cells in contrast to the non-methylated status of the parental cell lines.

## Materials and methods

### Cell cultures

MEL-DS19 and MEL-resistant (MEL-R) cells were cultured in Dulbecco’s modified Eagle’s medium (DMEM) supplemented with 10% fetal bovine serum (FBS) and 100 units/ml of penicillin and streptomycin (Gibco). Cell differentiation was induced by exposing logarithmically growing cell cultures to 5mM of HMBA. MEL-R cells were routinely cultured in the presence of the differentiation inducer. Hemoglobinized cells were monitored by determining the proportion of benzidine-staining positive cells (B+) of cell cultures. In order to analyze the epigenetic changes of the PU.1 locus, MEL-DS19 and MEL-R cells were grown in the absence or presence of 0,4 μM or 0,8 μM 5-Aza-2′-deoxycytidine (5-azaC, Sigma).

### PCR analysis

PCR experiments were performed with MEL-DS19 and MEL-R genomic DNA samples to sequence the viral–host DNA junction. PCR amplifications were performed using 200 μM of each nucleotide, 0.4 μM of sense and antisense primers and 1.25 units of Taq DNA Polymerase (Invitrogen). The PCR program consisted of an initial denaturation at 95°C during 3 min, followed by 30 consecutive cycles at 94°C during 45 s, annealing at 62°C during 30 s, extension at 72°C during 30s and a final extension at 72°C during 10 min. Long-range PCR (LR-PCR) was performed using the LongRange PCR Kit (Qiagen) and amplifications were performed using 500 μM of each nucleotide, 0.4 μM of sense and antisense primers and 2 units of LongRange PCR Enzyme Mix. The LR-PCR conditions comprised: denaturation at 93°C during 3 min, followed by 35 repeated cycles at 93°C during 15 s, annealing at 62°C during 30 s and extension at 68°C during 8 min. The following Sfpi-1/PU.1 and SFFV-specific primers were used: PU.1 Fw: 5′-TCCGCTCAAGACCAGGTC-3′; PU.1 Rv: 5′-CCATGTAGCCTTCTGAGT-3′, SFFV 1: 5′-AAGAACAGATGGTCCCCAGA-3′; SFFV 2: 5′-AAGGCACAGGGTCATTTCAG-3′; SFFV 3: 5′-AAAGAGCTCACAA CCCCTCA-3′; SFFV 4: 5′-GCCCAACGTTAGCTGTTTTC-3′; SFFV-a 5′-CAGAACCAGACGCAGGCGCA-3′; SFFV-b 5′-TCCACCATCATGGGGCTTCTCA-3′; SFFV-c 5′-TCCGCTCAAGACCAGGTC-3′; SFFV-d 5′-AGAGAGGTGAGAGTCATGCAATG-3′. PCR products were resolved on agarose gels and visualized by ethidium bromide staining. The *Sfpi-1/PU.1* and virus-specific primers were used for sequencing which was carried out by Secugen SL (CIB, Madrid).

### Gene expression analysis

Total RNA was isolated from 1 × 10^7^ cells using the TRIzol (GIBCO BRL) kit following the manufacturer’s instructions. For the semi-quantitative RT-PCR the reactions consisted of 5 μg of total RNA previously extracted from the MEL and MEL-R cells, which was reverse transcribed using the M-MLV reverse transcriptase (USB) as previously has been described (García-Sacristán et al. 2005). PCR amplifications were performed using 200 μM of each nucleotide, 0.5 μM of the sense and antisense primers plus 5 units of Recombi-Taq (LINUS). The amplification conditions encompassed: denaturation at 95°C during 2 min, followed by 30 cycles of 94°C during 1 min, annealing during 1 min was carried out at different temperatures depending on the primers used and extension was undertaken at 72°C during 1 min, with a final extension at 72°C during 5 min. The annealing temperatures were 55°C for the Shp1 primer pair and 65°C for the GAPDH. The following primers were used: Shp1 Fw 5′-CAGGATGGTGAGGTGGTTT-3′; Shp1 Rv 5 -CTCAAACTCCTCCC AGAAG-3′. For the quantitative real time PCR analysis RNA was isolated from 5 × 10^6^ cells using the RNeasy Mini Kit (Qiagen) as described by the supplier. RNA samples were reverse-transcribed using SuperScript® II Reverse Transcriptase (RT) (Invitrogen). First-strand cDNA was synthesized from 2.0 μg of total RNA using the Superscript II (Invitrogen) in a final volume of 20 μl with 0.5 μg of Oligo dT (Invitrogen), 20 units of SUPERase In RNase Inhibitor (Ambion) and 200 units of Superscript II reverse transcriptase. The reaction mixture was incubated at 42°C during 50 min. Quantitative real time PCR was carried out in iQ5 system (Bio-Rad). The reaction mixture of 20 μl consisted of 1X iQ SYBR Green Supermix (Bio-Rad), 1 μl cDNA and 0.2 mM of each primer. The PCR protocol consisted of: 95°C during 5 min, followed by 50 cycles of 95°C during 30s and 60°C during 30s. The following primers were used: PU.1 Fwd 5′-GGGATCTGACC AACCTGGA-3′ and PU.1 Rev 5′-AACCAAGTCATCCGATGGAG-3′. The relative gene-expression quantification method was used to calculate the amount of mRNA expression change according to the comparative C_t_ method using β-actin as an endogenous control. Final results were determined as follows: 2^-(ΔCt sample - ΔCt control)^, where the ΔC_t_ values of the control and the sample were determined by subtracting the C_t_ value of the target gene from the value of the β-actin gene. All experiments were performed in triplicate; differences in cell input were compensated by normalization against the β-actin expression levels.

### Bisulfite sequencing

Sodium bisulfite conversion was undertaken with the EZ DNA Methylation-Direct Kit (Zymo Research). The DNA bisulfite conversion was performed directly from 8 × 10^4^ cells according to the manufacturer’s instructions. Samples of 2 μl of bisulfite-treated DNAs were eluted in a 10 μl volume for each PCR reaction. Converted DNAs were amplified and sequenced by PCR using primers specific to the bisulfite-converted gDNA and the ZymoTaq™ DNA Polymerase (Zymo Research). The PCR protocol used was: 95°C during 10 min, followed by 35 cycles of 95°C during 30 s, 55°C during 30 s and finally 72°C during 40 s. The set of primers for bisulfite sequencing PCR (BSP) comprised: PU.1 bis DNA Fw 5′-GAAAGGAGATAAAATGTGGGAGAT-3′ and PU.1 bis DNA Rv 5′-CCAAATAATCCACTATTCTTTTAACCT-3′. The PCR products were separated and visualized in 1% agarose gels, followed by sequencing for methylation status evaluation. Sequencing was performed by Secugen SL (CIB, Madrid).

### Measurement of DNA methylation by pyrosequencing

Sodium bisulfite modification of 0.5 μg of genomic DNA isolated from MEL-DS19 and MEL-R was carried out with the EZ DNA Methylation Kit (Zymo Reserch) following the manufacturer’s protocol. Samples of 2 μl bisulfite-treated DNA were eluted in a 15 μl volume for each PCR reaction. The region of interest was amplified by PCR using primers specific to the bisulfite-converted gDNA. As a control, the efficiency of the bisulfite conversion was assessed using primers for the non-converted DNA sequence. Pyrosequencing was performed using the following primers: Fw 5′-AGTTTGGTAGTTTTGGGATTAAAG-3′; Rev 5′ [Btn] ATTTCTTCTCAATCCCCTCTAA-3′; seq 5′-AAATTTATTTTTAAAATTAGGGA-3′. Pyrosequencing reactions and methylation quantification were performed in a PyroMark Q24 System version 2.0.6 (Qiagen). Graphic representation of methylation values showed bars identifying CpG sites that represented methylation percentages. Assay design reports, which include the target sequence region and primer sequences, are provided as Additional file 1.

### Plasmid construction and DNA transfections

The PU.1-ER construct was generated by PCR using the Expand High Fidelity PCR System (Roche) to amplify the cDNA of PU.1 derived from MEL-DS19 cells. The primers contained BamHI recognition sites and consisted of the following sequences: PU.1-ER Fw 5′-GGGGCACCTGGTCCTGAG-3′ and PU.1-ER Rv 5′-CGCGGATCCGAGTGGGGCGGGAGGCG-3′*.* The expression vector producing the PU.1-ER fusion protein was constructed by subcloning the 869 bp BamHI fragment encoding the PU.1 cDNA into the pEBBpuro ER vector digested with the same enzyme. The inducible vector pEBBpuro GATA-1-ER (a generous gift from A. Skoultchi) was used to produce the conditionally active form of GATA-1. Stable transfectants of MEL-R were prepared as described previously (Vanegas et al. 2003). In short, recombinant DNA was introduced into exponentially growing MEL cells by lipofectine (Gibco) and after a 6 h incubation period cells were distributed into 96-well plates. The transfectants were selected and maintained in growth medium containing 5 μg/ml puromycin. Cell differentiation was tested by cell culture in the absence or presence of 5 mM HMBA with or without 10^-7^ M β-estradiol. Cell growth was measured on a daily basis by aliquot counting of the cultures with a Neubauer hemocytometer chamber.

### Antibodies and immunoblot analysis

MEL and MEL-R cells (1 × 10^7^) were pelleted, washed twice with cold PBS and lysed with Laemmli buffer (65 mM Tris–HCl pH 6.8, 10% glycerol, 5% 2-mercaptoethanol, 1% SDS) containing protease inhibitors (SIGMA). Proteins (20–50 μg) were resolved in a 10% SDS-polyacrylamide gel electrophoresis and transferred to PVDF membranes (Millipore). Primary antibodies included rabbit polyclonal anti-Sat1 antibody (1:1,000 Cell Signaling), rabbit polyclonal anti-Phospho-Sat1(Tyr 701) antibody (1:1,000 Cell Signaling), rabbit polyclonal anti-PU1 antibody (1:1,000; Santa Cruz) and mouse monoclonal anti-α-Tubulin antibody (1:10,000, Sigma).

## Results

### Mapping the SFFV integration site within the *PU.1* locus of MEL and MEL-R cell lines

**Figure 1:**
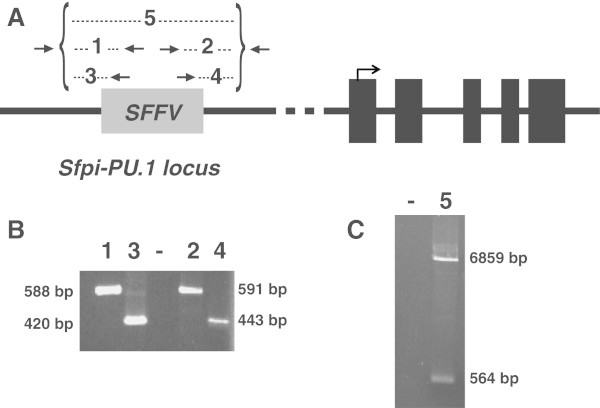
**PCR probe to confirm the SFFV integration site within the PU.1 locus. A)** Illustration of the multiple verification PCR amplifications using MEL genomic DNA as a template. Black arrows over the SFFV genome (gray box) indicate the pair of primers designed to amplify the SFFV-PU.1 junctions. Odd numbers 1 and 3 represent the primers used to identify the upstream integration junction and even numbers 2 and 4 represent the primers used for the downstream integration. Number 5 represents the long-range PCR (LR-PCR) used as a probe to confirm the complete SFFV integration. Black boxes correspond to the five exons of PU.1; the arrow above exon number one represents the initiation and direction of translation. **B)** Agarose gel electrophoresis performed using the primers schematized in **A)**. **C)** Agarose gel electrophoresis of the LR-PCR probe to confirm SFFV integration; both the wild type (564 bp) and the integrated allele (6,859 bp) are visualized.

**Figure 2:**
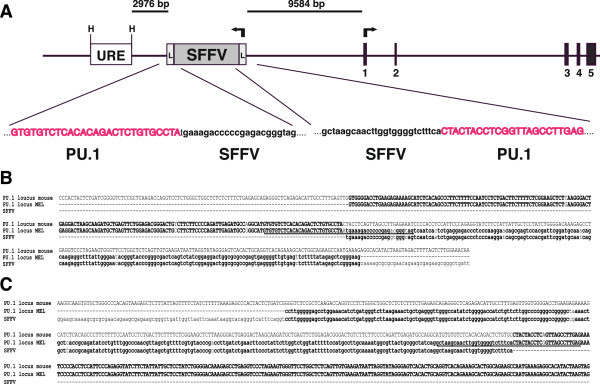
**Map of the PU.1 genomic region showing the location and orientation of the SFFV proviral integration in MEL-DS19 cells. A)** Diagram (drawn to scale) of the MEL-DS19 PU.1 genomic locus showing the location of a 3.4-kb HindIII (H) fragment referred to as the kb-14 URE (Upstream Regulatory Element) (Okuno et al. 2005), the SFFV genome is integrated downstream of the URE (gray box flanked by *white boxes* that symbolize the viral “long terminal repeats” (L)), and the five coding exons (black boxes) of the PU.1 locus. The sequences drawn below represent the upstream and downstream viral–host junctions, respectively. **B)** and **C)** Sequence alignment of the SFFV integration site displaying the PU.1 genomic sequences of mouse and of the MEL-DS19 cell line together with the SFFV genomic sequence. The PU.1 genomic sequence of the MEL-DS19 cell line corresponds to the upstream **(B)** and downstream **(C)** viral–host junction. Viral-host junctions as indicated in **A)** are underlined.

The Friend spleen focus-forming virus (SFFV) comprises a replication-defective retrovirus that in cooperation with the murine leukemia virus (MuLV) causes erythroleukemia in mice (revised in (Moreau-Gachelin 2006; Ruscetti 1999)). SFFV induces transformation of proerythroblasts by insertional mutagenesis into the *PU.1* locus which leads to the constitutive activation of the gene. Earlier studies have identified the SFFV proviral integration in a concrete region adjacent to the *PU.1* (also called *Sfpi-1* or *Spi-1*) gene (Moreau-Gachelin et al. 1988; Paul et al. 1989). More recently, integration of the SFFV was delimited to the URE located 14 kb upstream of the *PU.1* transcription start site (Okuno et al. 2005). Previously, we had used Southern blotting to show that SFFV integration occurs at a similar location in both MEL and MEL-R clones (Fernández-Nestosa et al. 2008). To determine the exact position of the SFFV insertion within the *PU.1* locus we employed a PCR approach using primer pairs of the proviral LTR (long terminal repeat), SFFV-genome specific and *PU.1*-specific sequences of upstream regions. After several attempts we ended up with primer pairs which amplified products derived from both of the putative SFFV-*PU.1* junction ends. Subsequently we used nested primers for a second round of PCR amplification followed by fragment isolation and sequencing (Figure 1A and B). After identification of both SFFV-*PU.1* junction ends, a Long range-PCR (LR-PCR) reaction was carried out using the reverse and forward *PU.1* primers (Figure 1C). Two bands of 6,854 bp and 564 bp, corresponding respectively to the integrated SFFV and the wild type alleles, were amplified in both cell lines (MEL and MEL-R). Results for MEL-R cells are shown in Additional file 1: Figure S1. Figure 2A represents the *PU.1* locus drawn to scale displaying the upstream localization of the SFFV genome. A total of 9,584 bp separate the closest SFFV long terminal repeat from the functional ATG of PU.1, while 2,976 bp divide the proviral distant long terminal repeat and the 3.4 kb HindIII-fragment containing the URE. Our results have clearly shown that the SFFV genome integration into the *PU.1* locus occurred outside of the URE. We have also confirmed that the provirus is integrated in an opposite transcriptional orientation as related to that of the PU.1 locus (Additional file 1: Figure S2).

### Reactivation of the silenced PU.1 locus of MEL-R cells by 5-azaC treatment

**Figure 3:**
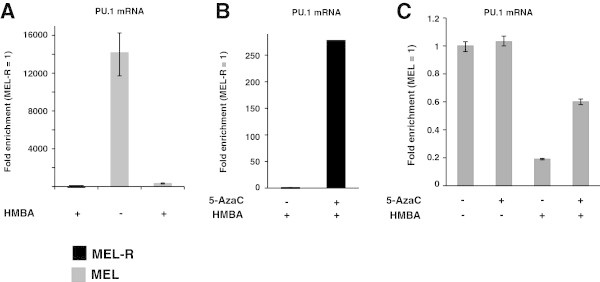
**Reactivation of the silenced PU.1 locus by 5-azaC treatment of MEL-R cells.** Gene expression analysis of PU.1 using quantitative real time PCR (qRT-PCR) of MEL DS19 and MEL-R cell lines after treatment with 5-azaC. **A)** Non-induced MEL and MEL-R cells. MEL-R cells were routinely cultured in the presence of HMBA (+). **B)** MEL-R cells cultured for 24 hr in the absence (-) or presence (+) of 0.4 μM 5-azaC. **C)** MEL DS19 cells cultured during 24 hr in the absence (-) or presence (+) of 0.4 μM 5-azaC and 5 μM HMBA. The magnitude of gene expression was calculated using the ∆∆Ct method. Each value is the average of three readings.

A major difference detected between leukemia HMBA-resistant cells and their parental MEL cells comprised the unexpected inactivation of PU.1 in MEL-R cell lines (Fernández-Nestosa et al. 2008). Furthermore, treatment with 5-Aza-2′-deoxycytidine (5-azaC) while not with trichostatin A (TSA) triggered PU.1 expression in all MEL-R clones. Since 5-azaC acts as an inhibitor of methylation maintenance (Christman 2002) it has been suggested that the activation of PU.1 expression in MEL-R is caused by a DNA methylation status change. We have performed in the present study a quantitative analysis regarding relative PU.1 expression in both resistant and parental cell lines as well as in cells after treatment with 5-azaC. Quantitative real-time RT-PCR (qRT-PCR) was first used to analyze the relative PU.1 mRNA expression in MEL and MEL-R (Figure 3A). We found that transcripts for PU.1 were expressed a several fold higher in MEL cells compared to the resistant cell lines. Cell exposure to 5-azaC caused a circa 200-fold increase of PU.1 mRNA expression in MEL-R cells (Figure 3B). On the contrary, a ~3 fold increase in PU.1 mRNA was observed during the course of MEL differentiation when cells were cultured in the presence of HMBA; no significant difference was detected in the non-induced (-HMBA) MEL cells (Figure 3C). Nevertheless, PU.1 mRNA levels of MEL-R cells treated with 5-azaC never reached those observed for MEL cells. These results have shown that PU.1 expression was boosted by 5-azaC application to MEL-R cells yet transcription activity was much lower in the non-induced and not differentiated MEL cells.

### Methylation status of CpG islands at the PU.1 promoter

**Figure 4:**
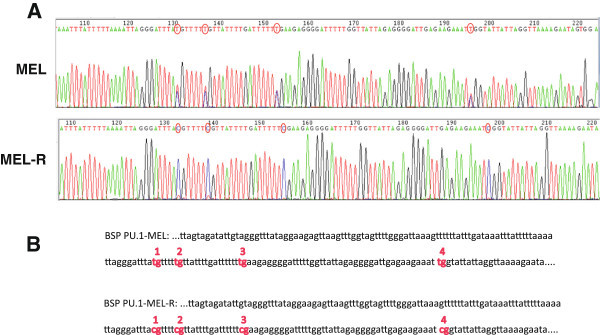
**Methylation status of the PU.1 promoter region. A)** DNA chromatogram of the PU.1 upstream region using bisulfite-treated genomic DNA derived from MEL and MEL-R cell lines. Red circles highlight the cytosines of the four CpG islands of the MEL-R cell line that changed to thymines in the MEL-DS19 cell line after bisulfite treatment. **B)** Methylation analysis of the PCR products of the bisulfite-treated genomic DNA from A). The CG sequences were numbered according to (Shearstone et al. 2011) and their methylation status was determined.

**Figure 5:**
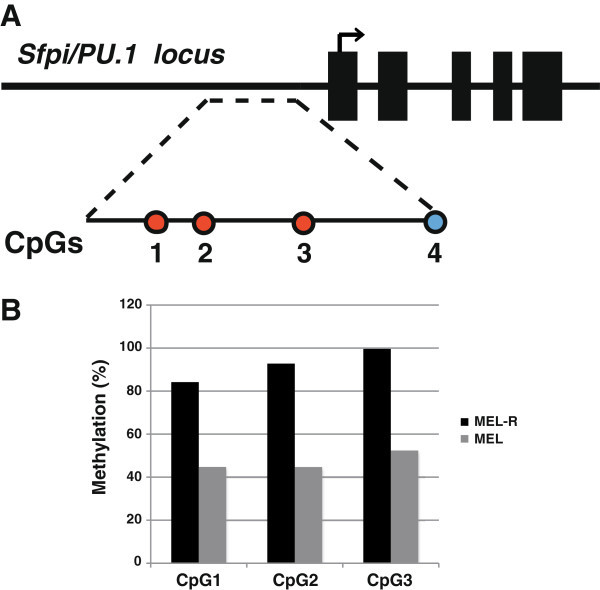
**DNA methylation status of the PU.1 locus regulatory region. A)** A genomic map (not drawn to scale) depicts the regions containing the analyzed CpGs. The CpG colored in blue represent PCR amplified islands which however were not detected by the sequencing primers. CpG colored in red represent sequenced islands. **B)** Individual CpG methylation percentages of MEL DS19 and MEL-R cell lines.

CpG islands consist of genomic regions characterized by a high content of CpG dinucleotides, usually unmethylated and associated to most known transcription starting sites (Jones 2012). Cytosine methylation in vertebrates is a common epigenetic mark associated to gene silencing. Our previous results have shown a PU.1 gene expression increases after treatment with 5-azaC in differentiated MEL cells, but in particular in MEL-R clones ((Fernández-Nestosa et al. 2008) and the present study). To explore changes in the methylation status of the PU.1 promoter that would eventually lead to DNA silencing we have used a bisulfite conversion followed by DNA sequencing of the PU.1 promoter region which comprises four CpG islands (Shearstone et al. 2011) (Figure 4). We first confirmed that all the sites of the non-CpG cytosine islands were displayed as thymines, which implied that the bisulfite-induced conversion of unmethylated cytosines to uracil in our samples was all-encompassing (Additional file 1: Figure S3). The analysis confirmed that in MEL-R cells all four CpG islands remained stably methylated, while in the MEL parental cell lines the conversion of the four cytosines (Cs) into thymines (Ts) revealed the unmethylated status. When we analyzed in detail the chromatogram sequence we observed some overlapping of Cs and Ts at individual peaks of CpG implying partial methylation. As differentiation proceeds under HMBA treatment a more pronounce overlapping is observed (Additional file 1: Figure S4). To quantify the DNA methylation levels we preceded to bisulfite pyrosequencing using the sets of primers for PCR amplification described by Shearstone et al. (2011) (Shearstone et al. 2011), which allow for the analysis of the first three CpG islands of the PU.1 promoter (Figure 5). The results showed that more than 80% of CpG1, CpG2 and CpG3 were methylated in MEL-R cells in contrast to only 40% methylation observed for the MEL progenitor cells. Taken together, these experiments confirmed that the PU.1 promoter is methylated in MEL-R cells, which moreover suggests that this methylation status might contribute to PU.1 silencing in these cell lines.

### 5-azaC treatment induces MEL-R cell differentiation in the presence of HMBA

**Figure 6:**
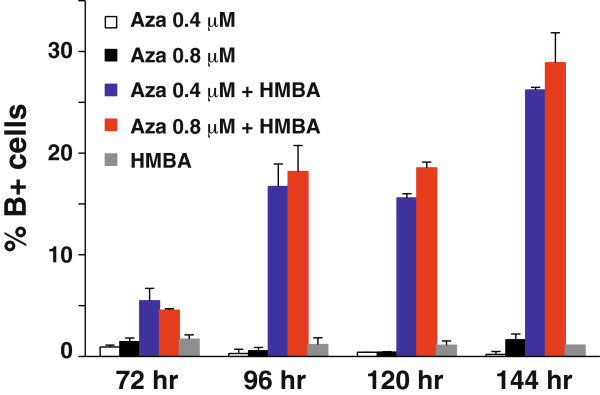
**Treatment with 5-azaC induces MEL-R cells to differentiate in presence of HMBA.** Percentage of differentiation of MEL-R cells was determined by the benzidine assay. Cells were cultured in the absence or presence of different concentrations of 5-azaC, with or without 5 mM HMBA. Each bar indicates the mean and standard deviation of three independent experiments.

We hypothesized that cell differentiation might follow demethylation as had previously been shown in MEL progenitor cells (unpublished results). To address the potential of 5-azaC to induce differentiation of MEL-R cultures, we treated cells with two different concentrations of 5-azaC (which do not compromise cell viability, unpublished results) in the presence or absence of 5mM HMBA. Percentage of cell differentiation was measured counting benzidine positive cells at different time points (Figure 6). The results clearly showed that 5-azaC alone was unable to induce cell differentiation given the insignificant proportion of B + cells present in the MEL-R cultures treated with 0.4 or 0.8 μM 5-azaC. On the contrary, when HMBA was used in combination with 5-azaC a substantial proportion differentiated reaching B + cells a proportion of about 30% after 144 hours of treatment. Altogether, these data have demonstrated that demethylation is necessary but not sufficient to overcome the differentiation blockage. However, removal of methylation marks would result in a receptive chromatin status and lead inducers such as HMBA, that by themselves have no effect on MEL-R differentiation ((Fernández-Nestosa et al. 2008) and Figure 6), to trigger cells to resume the differentiation program.

### Ectopic expression of PU.1 limits the proliferative capacity yet is not required to induce MEL-R cell differentiation

**Figure 7:**
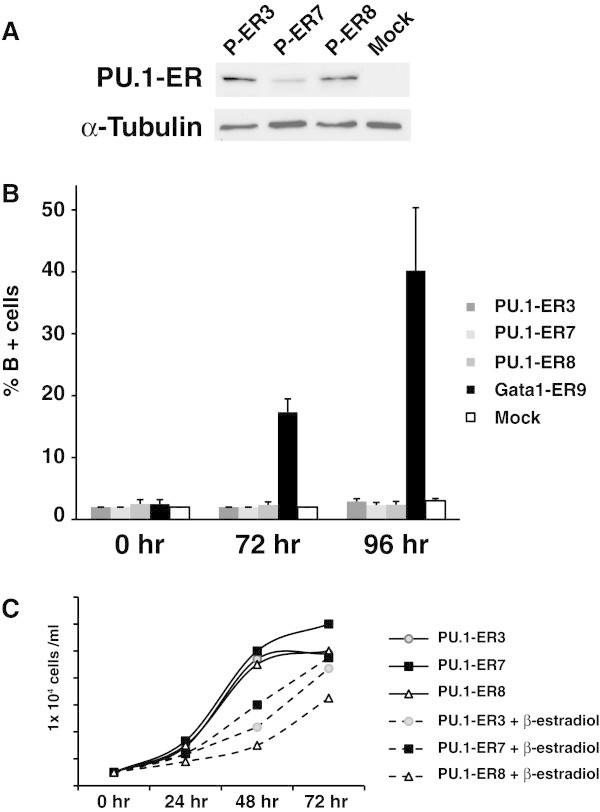
**Ectopic expression of PU.1 restricts the proliferative capacity but is unable to induce MEL-R cell differentiation. A)** Western blot analysis of lysates derived from MEL-R PU.1-ER transfectants stimulated with β-estradiol. Equal amounts of protein (50 μg) were fractionated by SDS-polyacrylamide gel electrophoresis and analyzed by immunoblotting with an anti-PU.1 antibody. Numbers above the panel correspond to different clones. **B)** Percentage of benzidine positive cells (B+) in PU.1 and GATA-1 stable transfectants activated by β-estradiol and cultured in the presence of 5 mM HMBA. Each bar indicates the mean and standard deviation of three independent experiments. **C)** PU.1-ER transfectants were cultured in the presence or absence of β-estradiol and cell counts were recorded at the indicated times.

Our results have shown that treatment with 5-azaC activated PU.1 expression and together with HMBA induced erythroid differentiation of MEL-R cultures (Figure 3 and (Fernández-Nestosa et al. 2008)). We hypothesized that PU.1 might be necessary for erythroleukemia cells to differentiate and therefore, restoring PU.1 function in MEL-R cells would bring back the induced-differentiation potential to these cell lines. In order to overexpress PU.1 we generated a stable transfection introducing vector containing a conditional PU.1 fused to the ligand-binding domain of the estrogen receptor (PU.1-ER) in order to carry out the transformation of MEL-R cells. Puromycin-resistant clones were isolated by a limited dilution and analyzed for the expression of the chimeric PU.1-ER protein (Figure 7A). We next looked for the ectopic PU.1 expression effect on the production of B + cells when transfectants were grown in the presence of β-estradiol and HMBA (Figure 7B). A conditional GATA1-ER clone (Fernández-Nestosa et al. 2008) was used as the control. The results have shown that forced expression of PU.1 is unable to trigger MEL-R cell differentiation. In all of the transfectant strains analyzed, PU.1-ER3, PU.1-ER7 and PU.1-ER8, the percentage of B + cells observed consisted of very low values (less than 1%) in line with the mock. On the contrary, cell growth was markedly reduced in all of the analyzed transfectants, with the reduction being more pronounced commencing 24 hours after activation of PU.1 (Figure 7C). These results are consistent with previous reports on MEL (Yamada et al. 1997) and other cell lines for which PU.1 was shown to induce growth arrest and apoptosis.

### MEL-R cells show similar expression levels of tyrosine phosphatase Shp-1 as those of the progenitor MEL cell lines

**Figure 8:**
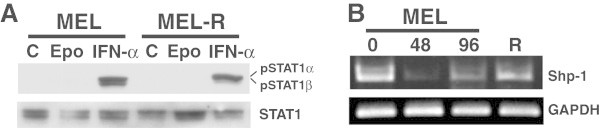
**STAT1 tyrosine phosphorylation analysis of MEL DS19 and MEL-R cell lines. A)** Non-stimulated MEL DS19 and MEL-R erythroleukemia cells (C) and stimulated with erythropoietin (100 U/ml) (Epo) or interferon α (IFN-α) (500 U/ml). Total cell lysates were immunoblotted with either anti-phospho-STAT1 (tyrosine 701) (upper panel) or anti-STAT1 antibody (lower panel). **B)** Comparison of Shp-1 expression by semi-quantitative RT-PCR of the parental MEL cells untreated (0) or treated with HMBA during 48 or 96 hours (48, 96) and of MEL-R cells (R). PCR products were normalized to GAPDH, separated by electrophoresis in a 1% agarose gel and stained with ethidium bromide.

Previous work in Dr. Ruscetti’s lab had shown that the inappropriate expression of PU.1 in SFFV-infected erythroid cells leads to increased tyrosine phosphatase Shp-1 levels which influence some signaling components of the Epo/EpoR pathway (revised in (Cmarik and Ruscetti 2010)). Particularly important was the block of the erythropoietin-induced Stat1/3 phosphorylation resulting in an obstruction of the DNA binding capacity, which in turn is crucial for erythroid differentiation (Nishigaki et al. 2006). Given that MEL-R differentiation blockage is independent of PU.1 overexpression, it is highly unlikely that the impediment to differentiate would reside in the inactivation of the Stat proteins through tyrosine dephosphorylation by SHP-1. In order to test whether the Stat1 inactivation occurred in MEL-R cell lines we performed a Western blot analysis using an anti-phospho-Stat1 antibody, which detects the phosphorylated tyrosine 701 of the α and β isoforms of Stat1 (Figure 8A). We distinguished two bands of 84 and 91 kDa corresponding to the α and β Stat isoforms, respectively, after stimulation with IFN-α of MEL and MEL-R cell lines. On the contrary, we failed to detect phosphorylated Stat1 neither before nor after treatment with Epo. We also compared the phosphatase Shp-1expression levels of the MEL-R and MEL differentiation- induced cell lines (Figure 8B). We found that Shp-1 was highly expressed in the non-induced MEL cells (0 hr) and also in the MEL-R cells, yet rapidly declined as the cells underwent differentiation in response to HMBA (48 and 96 hr). Altogether, these results suggest that although Shp-1 could be related to the differentiation blockage of MEL-R cells, nonetheless, it is a PU.1-independent process.

## Discussion

### SFFV integration within the *PU.1* locus of MEL and MEL-R cell lines

PU.1 proviral insertion is a major event that occurs during the second stage of the Friend disease and which leads to a block of the erythroid differentiation program. It was initially recognized that SFFV integrates upstream of the PU.1 transcriptional start site (Moreau-Gachelin et al. 1989; Paul et al. 1989) and later on the target for SFFV integration was precisely located at -14 kb of the URE in a non-conserved 500-bp spacer, lying exactly between two highly conserved homologous regions (Okuno et al. 2005). The mechanism by which SFFV induces permanent PU.1 expression in erythroleukemia cells was suggested to rely on the disruption of the URE at the non-homologous spacer. This interruption might prevent the 5′ distal region to down-regulate PU.1 expression, while the 3′ proximal element constitutively activates PU.1 transcription. In this study, we have identified, at the nucleotide level, the exact location of the SFFV integration site within the PU.1 locus of erythroleukemia cells. We provide proof that the integration site resides outside of the URE, concretely 2,976 bp apart, which therefore excludes the hypothesis of the negative/positive regulation of the URE 5′ proximal/3′ distal regions, respectively. Instead, it seems that the SFFV integration might block a negative regulation of the URE on the PU.1 promoter which together with the transcriptional enhancer elements supplied by the SFFV long terminal repeats (LTR) result in an unsolicited activation of the gene. On the other hand, as was predicted in our previous study (Fernández-Nestosa et al. 2008), the SFFV insertion of the resistant cell lines (MEL-R) occupied an identical position compared to the parental cell lines. This observation rules out the possibility that PU.1 silencing in MEL-R cells may well be a consequence of either the absence of SFFV or that proviral integration might have occurred at a different spot.

These findings are focused on the MEL-DS19 cell line derived from the MELC 745A strain, which in turn derives from an original Charlotte Friend’s lab strain (Ohta et al. 1976). Therefore, these results do not preclude the integration of SFFV at a different site of the PU.1 locus in other host genomes. Many models for the selection of integration-sites by a retrovirus favor their integration near to DNase I-hypersensitive sites characterized by an open chromatin conformation (Lewinski et al. 2006). Genome mapping has allowed the detection of putative SFFV integration sites extended over several kilobases upstream of the PU.1 coding region (Moreau-Gachelin et al. 1988; Paul et al. 1991). Proviral insertion at the 5′ end of the PU.1 locus has also been described in leukemia stem cells infected with the Friend virus (Hegde et al. 2012; Hasegawa et al. 2005). In this study we have identified and sequenced the genome-viral junction region of MEL-DS19 and MEL-R clones and plan to further extend a similar approach to other SFFV-infected cell lines. Finally, novel cis-regulatory elements outside of the URE, about 10kb upstream of the PU.1 transcriptional start site, have recently been described (Zarnegar et al. 2010). These elements can simultaneously act as enhancers in myeloid cells and repressors in T cells and sometimes have been described to neutralize the continuous URE enhancer function. These newly characterized elements overlap with the SFFV integration site described in this report and suggest other possible mechanisms that can mediate PU.1 regulation.

### Methylation suppressed PU.1 expression of MEL-R cells

In this study we have demonstrated that 5′-CpG-3′ dinucleotides of the PU.1 promoter region are highly methylated in MEL-R cells, but not in the parental MEL-DS19 cell line. DNA methylation of CpG dinucleotides comprises one of the best characterized epigenetic marks that condition gene expression, usually producing a repressive transcription status with consequent gene silencing (Jones 2012). Analysis of the four CpG islands of the PU.1 promoter (Shearstone et al. 2011) showed a high level of methylation in MEL-R cells compared to the MEL parental cell lines. This hypermethylation could be responsible for the PU.1 inactivation of the resistant clones. Accordingly, reversal of this hypermethylated status caused by treatments with 5-azaC, a known antagonist of DNA methylases (Christman 2002), that have been reported to restore PU.1 expression (Amaravadi and Klemsz 1999; Tatetsu et al. 2007) induced the differentiation of MEL-R cell cultures. On the other hand, methylation of PU.1 in the MEL-DS19 cells was significantly lower than in MEL-R cell lines and the adding of 5-azaC to the cultures produced only minor alterations. The HMBA-induced differentiation did not modify the methylation status of PU.1 in the parental cell lines, as would have been expected based on the gradual silencing of PU.1 through erythroid differentiation. This truly represents a paradox as methylation prevents PU.1 expression in MEL-R cells leading to gene silencing and differentiation blockage. However, the gradual silencing of PU.1 during HMBA-induced erythroid differentiation has been reported not to consist of a methylation-related process (Shearstone et al. 2011). It has recently been reported that global DNA demethylation occurs continuously during mouse erythropoiesis *in vivo*, a process which is also associated to DNA replication and has been suggested to take place during differentiation of most somatic cells. The observed MEL-DS19 cell line PU.1 methylation status harmonizes with this model, even in the case of the HMBA-induced differentiation for which methylation of PU.1 is limited. On the contrary, PU.1 methylation of MEL-R clones follows the classical methylation concept as a robust mechanism for gene silencing. After methylation mark removal, PU.1 expression is restored and MEL-R cells become receptive to HMBA. This places emphasis on another controversy concerning the role of PU.1 in erythroleukemia cells. Differentiation blockage in murine erythroleukemia cells has been attributed to the inopportune expression of PU.1 (Rao et al. 1997). Erythroid differentiation is also partially blocked in PU.1 transgenic proerythroblasts (Moreau-Gachelin et al. 1996). However, previous findings have indicated a requirement of PU.1 expression for erythroid differentiation, Rosenbauer and coworkers demonstrated that the gradual activity reduction of PU.1, rather than the complete loss, can induce acute myeloid leukemia (AML) in mice (Rosenbauer et al. 2004). Two recent reports (Wontakal et al. 2011; Ridinger-Saison et al. 2012), on the other hand, have revealed the complexity of the pathways regulated by PU.1 in several hematopoietic lineages. PU.1 level differences are crucial for cell identity and even low concentrations of the protein can promote ubiquitous cellular functions (Wontakal et al. 2011). Altogether, these findings suggest that PU.1 is necessary for erythroid commitment, but contrasts with the downregulation requirement for terminal differentiation (Atar and Levi 2005; Rao et al. 1997; Yamada et al. 1997). PU.1 expression is abolished in the HMBA-resistant erythroleukemia cells and yet these cells are unable to differentiate (this study and (Fernández-Nestosa et al. 2008)). Low PU.1 levels might be necessary for erythroleukemia cells to differentiate, as probably is the case of MEL-DS19 cells. MEL-R cell lines, on the contrary, had a completely blocked (methylated) PU.1 expression and had lost their ability to respond to HMBA, a process that we have shown to be reversible by demethylating agents. We also observed that the expression of PU.1 driven by a conditional estrogen-activated element led to cell growth arrest and apoptosis, in agreement with earlier findings of MEL, K562 leukemia and myeloma cell lines (Aoyama et al. 2012; Tatetsu et al. 2007; Yamada et al. 1997). However, the ectopic expression of PU.1 could not have triggered the differentiation of MEL-R cells. It is most likely that additional factors, essential for erythroid differentiation, might have also been epigenetically silenced in resistant cell lines.

### STAT-1 phosphorylation blockage of MEL-R cells was independent of PU.1 expression

In addition to the ability of PU.1 to block erythroid terminal differentiation by antagonism with GATA-1, the locus mechanism of action may also apply to the activation of SHP-1. Previous work had already shown that high levels of SHP-1 interfered with STAT1 phosphorylation, thus preventing the binding activity and blocking erythroid differentiation in SFFV-transformed erythroleukemia cell lines (Nishigaki et al. 2006). It has also been shown that SHP-1 expression is significantly reduced in PU.1^-/-^ erythroid cells (Fisher et al. 2004). This data suggests that high PU.1 levels block differentiation signals although in an indirect way. We have observed that in MEL-DS19 cells, STAT1 α and β are neither tyrosine phosphorylated before nor after stimulation with erythropoietin (Epo). In addition, we also showed that SHP-1 was highly expressed in parental cell lines; nonetheless the expression was significantly reduced during the HMBA-induced differentiation. Unexpectedly, we have also noticed a failure of STAT1 to become phosphorylated in Epo-stimulated MEL-R cells in which PU.1 expression was completely abolished by DNA methylation. HMBA-resistant cell lines also showed a high SHP-1 expression. These results strongly suggest that in MEL-R cells the blocking of the STAT1 DNA binding activity is regulated by a different mechanism.

## Electronic supplementary material

Additional file 1 Figure S1: PCR confirmation of the SFFV integration site at the PU.1 locus in MEL-R cells. **A)** Illustration of PCRs performed using MEL-R genomic DNA as a template (see legend of Figure 1 details). **B)** Upstream and downstream confirmation PCRs. **C)** LR-PCR confirmation using MEL and MEL-R genomic DNA. **Figure S2.** Orientation of the SFFV provirus relative to the transcriptional direction of the PU.1 gene. **A)** Schematic drawing of the PU.1 locus showing the potential transcriptional orientation of the SFFV genome relative to the PU.1 transcript. Horizontal arrows indicate primers designed to confirm the direction of proviral integration. Arrows above exon 1 of PU.1 and above the SFFV genome indicate the transcriptional orientation. **c** and **d** represent upstream and downstream primers, respectively, of the PU.1 locus. **a** and **b** indicate the SFFV genome specific primers. **B)** and **C)** PCR products obtained using different combinations of PU.1 and SFFV primers in MEL **(B)** and MEL-R **(C)**; **cb** amplified a 1,765 bp and **ad** a 1,744 bp fragment, respectively. **Figure S3.** Specific demethylation at CpG dinucleotides of the PU.1 upstream regulatory region of MEL-DS19 and MEL-R. **A)** Genomic region from the PU.1 upstream locus that includes the bisulfite converted region (bold) with the four CpG dinucleotides (red) as described in (Shearstone et al. 2011). Underlined sequences correspond to the primers used for pyrosequencing. **B)** Alignment of a 187 bp fragment amplified after bisulfite conversion in MEL-DS19 (BSP-MEL) and MEL-R (BSP-MEL-R); the same fragment of the mouse genome (gDNA) is included as a comparison. In red, unchanged Cs after bisulfite, in blue Cs changed to Ts. **Figure S4.** Methylation status of the PU.1 promoter region in HMBA treated cells during 48 and 96hr, respectively. Red circles highlight the cytosines of the four CpGs. N represents the positions where the C and T peaks overlapped. (PDF 2 MB)
