# Supplementary material for: DNA methylation-mediated silencing of PU.1 in leukemia cells resistant to cell differentiation
Source: Springerplus. 2013 Aug 21;2:392. doi: 10.1186/2193-1801-2-392 (PMC3758488; doi:10.1186/2193-1801-2-392)
Supplement: Supplementary file 1 — Additional file 1 Figure S1: PCR confirmation of the SFFV integration site at the PU.1 locus in MEL-R cells. A) Illustration of PCRs performed using MEL-R genomic DNA as a template (see legend of Figure 1 details). B) Upstream and downstream confirmation PCRs. C) LR-PCR confirmation using MEL and MEL-R genomic DNA. Figure S2. Orientation of the SFFV provirus relative to the transcriptional direction of the PU.1 gene. A) Schematic drawing of the PU.1 locus showing the potential transcriptional orientation of the SFFV genome relative to the PU.1 transcript. Horizontal arrows indicate primers designed to confirm the direction of proviral integration. Arrows above exon 1 of PU.1 and above the SFFV genome indicate the transcriptional orientation. c and d represent upstream and downstream primers, respectively, of the PU.1 locus. a and b indicate the SFFV genome specific primers. B) and C) PCR products obtained using different combinations of PU.1 and SFFV primers in MEL (B) and MEL-R (C); cb amplified a 1,765 bp and ad a 1,744 bp fragment, respectively. Figure S3. Specific demethylation at CpG dinucleotides of the PU.1 upstream regulatory region of MEL-DS19 and MEL-R. A) Genomic region from the PU.1 upstream locus that includes the bisulfite converted region (bold) with the four CpG dinucleotides (red) as described in (Shearstone et al. 2011). Underlined sequences correspond to the primers used for pyrosequencing. B) Alignment of a 187 bp fragment amplified after bisulfite conversion in MEL-DS19 (BSP-MEL) and MEL-R (BSP-MEL-R); the same fragment of the mouse genome (gDNA) is included as a comparison. In red, unchanged Cs after bisulfite, in blue Cs changed to Ts. Figure S4. Methylation status of the PU.1 promoter region in HMBA treated cells during 48 and 96hr, respectively. Red circles highlight the cytosines of the four CpGs. N represents the positions where the C and T peaks overlapped. (PDF 2 MB) [file 40064_2013_469_MOESM1_ESM.pdf]

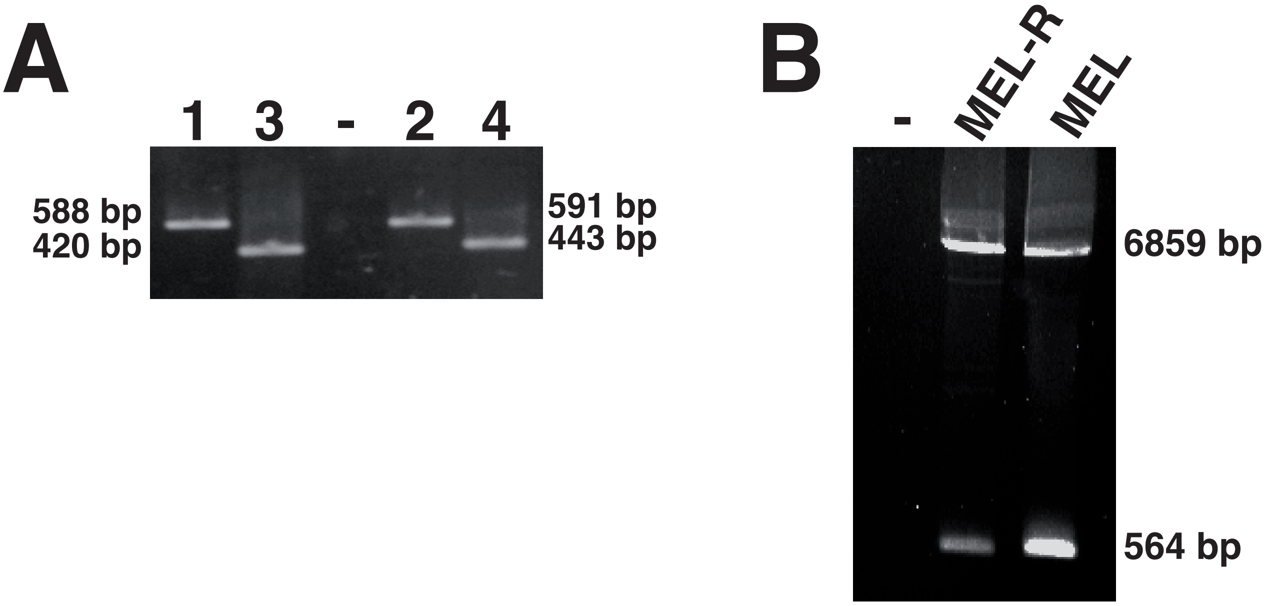

Suppl.Figure 1

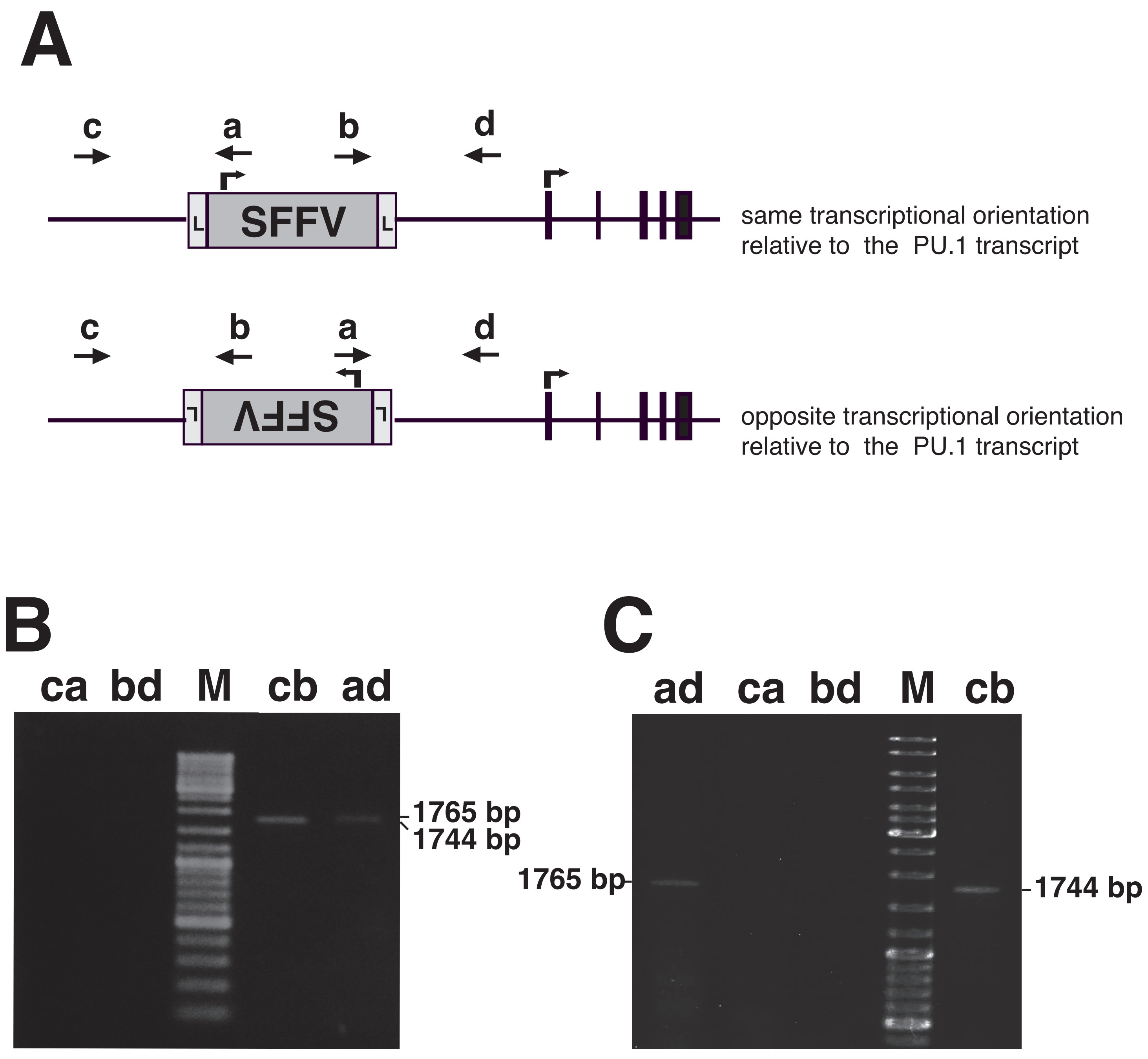

Suppl.Figure 2

A

....tctagatcgtaacgttcaagggttggagaaaccgggagaactgggctgggaacaatgtccttagggatctga
caccagctgtggtacttagctgggaggggaggggatcagaagccaatgtccttcccaattggaagggaagcccact
tgagaagtctggaaaggagataaaatgtgggagacccagtgtctactgttggccctaattggcttaggacgat**CTA**
GCAGATATTGCAGGGCCTACAGGAAGAGCCAAGCCTGGCAGCCTTGGGACCAAAGTCCTCCACCTGACAAATTCAC
CCTCAAAATTAGGGATCCACGCTCTCGCTATTCTGACCCCCCGAAGAGGGGACTCTTGGCCACCAGAGGGGACTGA
GAAGAAATCGGTATTATTAGGCTAAAAGAACAgtggactacttcagcaaggcctagcgaccggagcagcagaagcc
ttccatggtagtgctagcctttctccctcccagccccagtttcctctgggcaggggcctggcccctgtcttccca
gggctgccctttgagaaccacctgccccagccggccagagacttcctgtagcgcaagagatttatgcaaacgggct
ggggcggtgatgtcaccccaaggggactatctcccagtggcaggcccttcgataaaatcaggaacttgtgctggcc
ctgcaatgtcaagggagggggctcacccagggctcctgtagctcagggggcaggcctgagccctgcgtctgaccca
cgaccgtccagtcccccgacggggcacctggtcctgaggggggatccgccttgatccccaccgaagcaggggatctg
accaacctggagctcagctgg***atg***ttacagg.....

B

```
  !
BSP-MEL        1    TTAGTAGATATTGTAGGGTTTATAGGAAGAGTTAAGTTTGGTAGTTTTGGGATTAAAGTT   60
                    ||||||||||||||||||||||||||||||||||||||||||||||||||||||||||||
BSP-MEL-R      1    TTAGTAGATATTGTAGGGTTTATAGGAAGAGTTAAGTTTGGTAGTTTTGGGATTAAAGTT   60
                     ||| |||||||| ||||  || ||||||||  |||  ||| ||  ||||||  ||||| 
gDNA-sequence  1    CTAGCAGATATTGCAGGGCCTACAGGAAGAGCCAAGCCTGGCAGCCTTGGGACCAAAGTC   60


BSP-MEL        61   TTTTATTTGATAAATTTATTTTTAAAATTAGGGATTTATGTTTTTGTTATTTTGATTTTT  120
                    |||||||||||||||||||||||||||||||||||||| ||||| |||||||||||||||
BSP-MEL-R      61   TTTTATTTGATAAATTTATTTTTAAAATTAGGGATTTACGTTTTCGTTATTTTGATTTTT  120
                     |  |  ||| ||||| |   | ||||||||||||  | | ||| | ||||| ||     
gDNA-sequence  61   CTCCACCTGACAAATTCACCCTCAAAATTAGGGATCCACGCTCTCGCTATTCTGACCCCC  120


BSP-MEL        121  TGAAGAGGGGATTTTTGGTTATTAGAGGGGATTGAGAAGAAATTGGTATTATTAGGTTAA  180
                     |||||||||||||||||||||||||||||||||||||||||| ||||||||||||||||
BSP-MEL-R      121  CGAAGAGGGGATTTTTGGTTATTAGAGGGGATTGAGAAGAAATCGGTATTATTAGGTTAA  180
                     |||||||||| | ||||  |  |||||||| ||||||||||| |||||||||||| |||
gDNA-sequence  121  CGAAGAGGGGACTCTTGGCCACCAGAGGGGACTGAGAAGAAATCGGTATTATTAGGCTAA  180


BSP-MEL        181  AAGAATA   187
                    |||||||
BSP-MEL-R      181  AAGAATA   187
                    ||||| |
gDNA-sequence  181  AAGAACA   187
```

Suppl.Figure 3

MEL 48h

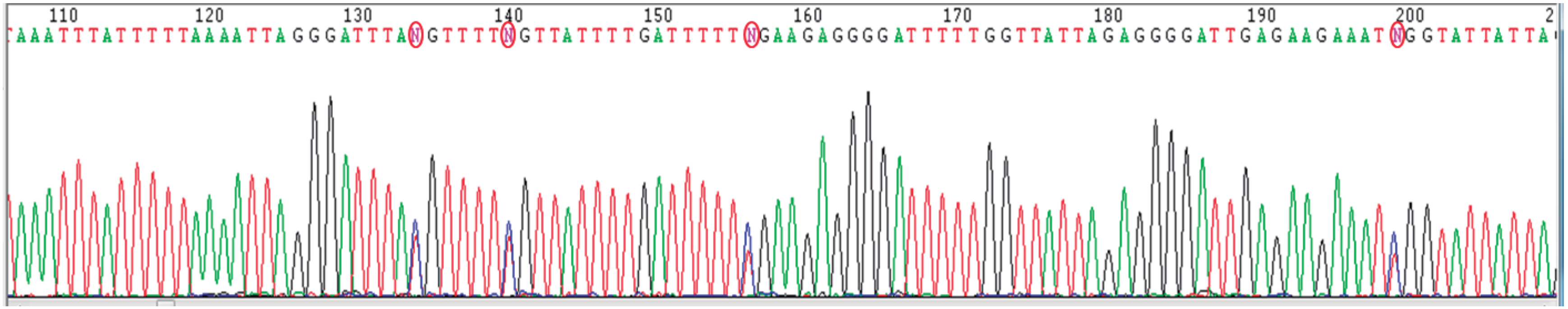

MEL 96h

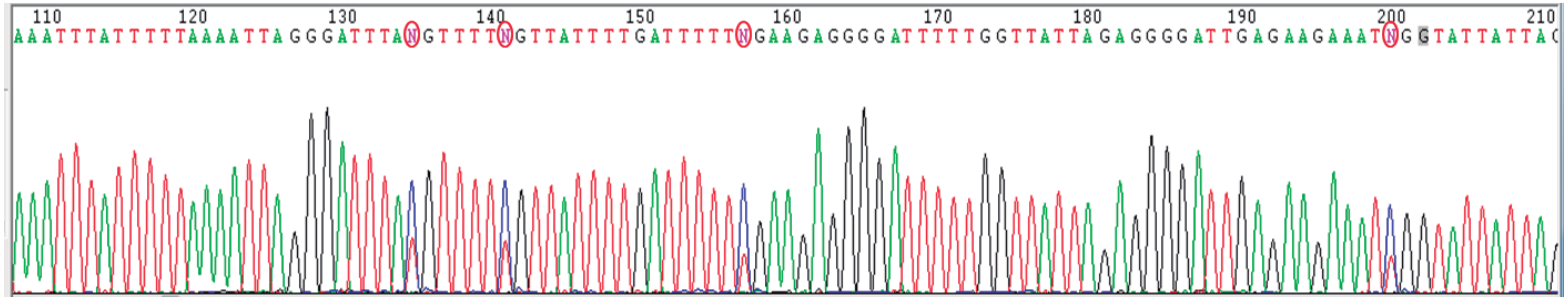

Suppl.Figure 4

Legends of Supplementary Figures

Supplementary Figure 1. PCR confirmation of the SFFV integration site at the PU.1 locus in MEL-R cells. A) Illustration of the multiple confirmatory PCRs performed using MEL-R genomic DNA as a template. Odd numbers 1 and 3 represent the primers used to identify the upstream integration junction and even numbers 2 and 4 represent the primers used to recognize the downstream integration. B) Agarose gel electrophoresis of the upstream and downstream confirmation PCRs. C) Agarose gel electrophoresis of the LR-PCR confirmation using MEL DS19 and MEL-R genomic DNA. Note the presence of both the wild type (564 bp) and the integrated allele (6,859 bp).

Supplementary Figure 2. Orientation of the SFFV provirus relative to the transcriptional direction of the PU.1 gene. A) Schematic drawing of the PU.1 locus showing the potential transcriptional orientation of the SFFV genome relative to the PU.1 transcript. Horizontal arrows indicate sets of primers designed to confirm the direction of proviral integration. Arrows above exon 1 of PU.1 and above the SFFV genome indicate the transcriptional orientation. **c** and **d** represent upstream and downstream primers, respectively, of the PU.1 locus. **a** and **b** indicate the SFFV genome specific primers. B) and C) Agarose gel electrophoresis of PCR products obtained using different combinations of PU.1 and SFFV primers in MEL (B) and MEL-R (C); **cb** amplified a 1,765 bp and **ad** a 1,744 bp fragment, respectively.

Supplementary Figure 3. Specific demethylation at CpG dinucleotides of the PU.1 upstream regulatory region of MEL-DS19 and MEL-R. A) Genomic region from the PU.1 upstream locus that includes the bisulfite converted region (bold) with the four CpG dinucleotides (red) as described in (Shearstone et al. 2011). Underlined sequences correspond to the primers used for pyrosequencing; the "atg" transcription initiation codon is indicated. B) Alignment of a 187 bp fragment amplified after bisulfite conversion in MEL-DS19 (BSP-MEL) and MEL-R (BSP-MEL-R); the same fragment of the mouse genome (gDNA) is is included as a comparison. In red, unchanged Cs after bisulfite, in blue Cs changed to Ts.

Supplementary Figure 4. Methylation status of the PU.1 promoter region during HMBA induced differentiation of MEL cells. DNA chromatogram of the PU.1 upstream region using bisulfite-treated genomic DNA derived from MEL cells treated with HMBA during 48 and 96hr, respectively. Red circles highlight the cytosines of the four CpGs. N represents the positions where the C and T peaks overlapped.
